# Overcoming Barriers to Cervical Screening Among Pacific Women: A Narrative Review

**DOI:** 10.1089/heq.2018.0076

**Published:** 2019-02-14

**Authors:** Georgina S. McPherson, Peggy Fairbairn-Dunlop, Deborah Payne

**Affiliations:** ^1^ Department of Clinical Sciences, Faculty of Health and Environmental Sciences, Auckland University of Technology, Auckland, New Zealand.; ^2^ Department of Obstetrics and Gynaecology, Colposcopy Clinic, Waitemata District Health Board, Waitakere Hospital, Auckland, New Zealand.; ^3^ Institute of Public Policy, Auckland University of Technology, Auckland, New Zealand.; ^4^ Centre for Midwifery and Women's Health Research, Faculty of Health and Environmental Sciences, Auckland University of Technology, Auckland, New Zealand.

**Keywords:** barriers, cervical screening, colposcopy, facilitators, Pacific women

## Abstract

**Purpose:** This narrative review explores the barriers and facilitators for Pacific women accessing the cervical screening pathway. Despite organized cervical screening in New Zealand, Pacific women still face significant health disparities in regard to cervical cancer incidence and mortality and access to colposcopy services. Providing a narrative synthesis of the available literature examining Pacific women and the barriers and facilitators to the cervical screening pathway may provide some insight into the provision of primary and secondary health services for Pacific women.

**Methods:** Four electronic databases were searched for articles published between January 1990 and June 2017 and included bibliographies of key journal articles and gray material. A narrative review and synthesis were undertaken of qualitative, quantitative, and mixed methods research.

**Results:** The literature is focused on the cervical screening aspect of the cervical screening pathway. There was a paucity of literature that examines the barriers and facilitators Pacific women experience accessing colposcopy services. Barriers to cervical screening for Pacific women are multifaceted and interrelated. Factors such as culture, fear, practical issues, health care experiences, and knowledge/education influence screening practices. Facilitators to cervical screening are also multifaceted and included knowledge, health care experience, culture, and practical issues. Culturally tailored approaches improve access to cervical screening for Pacific women.

**Conclusion:** Understanding Pacific women's experiences, facilitators, and barriers to the cervical screening pathway is essential in assisting health care professionals, policy makers, and funders provide culturally appropriate services. Further research is required to examine Pacific women's experiences of navigating colposcopy services and the interface between primary and secondary care services.

## Introduction

Cervical cancer is primarily a preventable disease. However, worldwide it is one of the leading causes of cancer mortality in women. Cervical cancer deaths number 266,000 every year and mainly occur in low- to middle-income countries where there is limited access to organized cervical screening programs.^1^ The primary cause of cervical cancer is due to persistent infection with oncogenic human papilloma virus (HPV). HPV infection is mostly transient in nature, although when persistent, it can cause precancerous changes on the cervix. If these changes are left untreated, they can develop into a cancer.^1,2^

Cervical screening plays an essential component in reducing cervical cancer incidence and mortality. Since the introduction of the National Cervical Screening Programme (NCSP) in New Zealand in the 1990s, there has been a significant decrease in the incidence and mortality of cervical cancer.^3^ While cervical screening coverage has improved for Pacific women in New Zealand, there are still significant inequities for them in regard to cervical cancer incidence and mortality.^4^ Pacific women are more likely to die of cervical cancer compared with European women. In 2012, cervical cancer mortality rates in Pacific women were reported as 4.6 per 100,000 compared with 1.6 per 100,000 in European/other women (age standardized). Cervical cancer incidence was 9.4 per 100,000 compared with 6.0 per 100,000, respectively.^3^

While cervical screening coverage is a key element of cervical cancer prevention, retrospective studies of women diagnosed with cervical cancer have demonstrated that between 9% and 17% of women had delayed follow-up and treatment following a high-grade cervical smear.^5–7^ Pacific women are less likely to access colposcopy services following a high-grade smear abnormality. Nearly a quarter of Pacific women referred with an high-grade smear abnormality have a delay of more than 90 days for their initial colposcopy compared with 9.9% for European/other women.^8^

There is literature exploring the interplay of cultural beliefs and practices on Pacific women's access to health care generally; understanding how that relates to the cervical screening pathway has not been explored in a systematic or narrative review. The importance of culture and practices is a key element in engaging Pacific women.^9,10^

For cervical screening programs to be successful, it is essential that high rates of screening coverage are achieved. This must also be accomplished with the follow-up and treatment of cervical abnormalities.^11–13^ Examining the literature may provide insights in reducing barriers and identifying facilitators for Pacific women navigating the cervical screening pathway: from screening to treatment.

## Methods

This narrative review and synthesis aim to identify and explore available literature on the barriers and facilitators to Pacific women accessing the cervical screening pathway. It was undertaken because no previous systematic or narrative review has been undertaken on the topic. Given the disparities Pacific women face in New Zealand, it is a timely opportunity to examine the current literature to identify any gaps and potential solutions.

A narrative review and synthesis approach was used to synthesize a range of varied studies in a structured approach. A narrative review provides the opportunity to systematically bring together a range of literature to provide an overview of the current literature on a topic incorporating a range of research methodologies. The narrative literature review approach is not so focused on scientific rigor as used in a systematic review but is interested in the narrative evidence on a topic.^14,15^ This narrative review aims to draw on themes identified in the literature across a number of studies. While there are limitations, narrative reviews provide a useful narrative on a particular issue in the literature where there is limited information.^14,15^

A search strategy was utilized to identify published and unpublished research on the barriers and facilitators to Pacific women accessing the cervical screening pathway. It was undertaken using the keywords identified in Table 1.

**Table 1.** Key Search Terms

1	Pacific women
2	Pacific Island women
3	Polynesian women
4	Barriers
5	Facilitators
6	1 and cervical smears/pap screening
7	2 and cervical smears/pap screening
8	3 and cervical smears/pap screening
9	1–4 and cervical smears/pap screening
10	1–3 and 5 and cervical smears/pap screening
11	1 and human papilloma virus
12	2 and human papilloma virus
13	3 and human papilloma virus
14	1 and cervical cancer
15	2 and cervical cancer
16	3 and cervical cancer
17	1–4 and cervical cancer
18	1–3 and 5 and cervical cancer
19	1 and colposcopy

A systematic electronic search was performed using four databases using the keyword search terms for literature published between January 1990 and June 2017. The databases searched were CINAHL, EBSCOhost, Cochrane library, and Scopus. A search of the Internet for gray literature was undertaken and bibliographies of full-text journal articles identified were hand searched for additional articles.

To determine the extent of the existing literature, all study types were included in the review, including qualitative, quantitative, and mixed methods studies. The criteria for inclusion were studies that focused on the barriers and facilitators to Pacific women accessing the cervical screening pathway. Studies were excluded where the results could not be directly attributed to Pacific women.

All studies identified from the database searches were saved with abstracts. Any duplicates were removed. Each abstract was assessed to determine if the article met the selection criteria. The relevant full articles were retrieved and then assessed to judge whether they met the eligibility criteria. Bibliography searches of the retrieved articles were undertaken and further articles of interest were identified. An Internet search of relevant gray literature was undertaken.

**FIG. 1.**
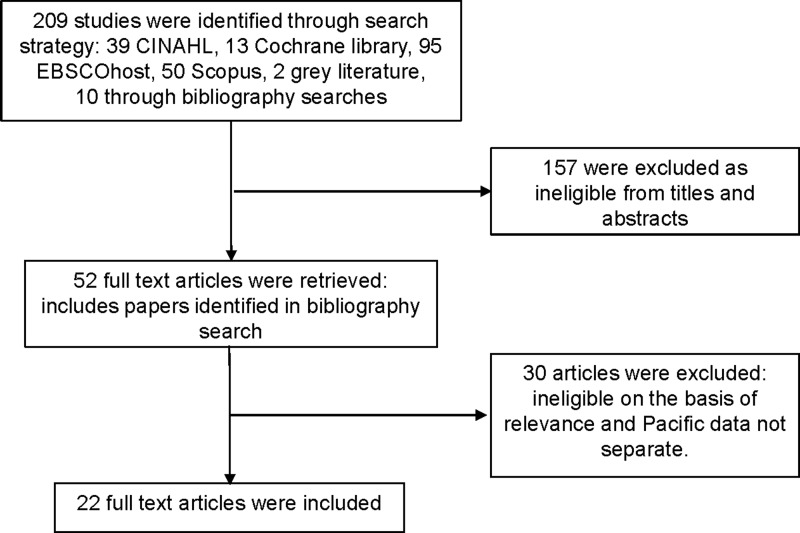
Search process flow chart.

The search strategy identified 285 studies, 76 duplicate entries were excluded leaving 209 studies (39 CINAHL, 13 Cochrane library, 95 EBSCOhost, 50 Scopus, 2 gray literature studies, and 10 bibliography searches). Forty-two full-text articles were retrieved. An assessment of the retrieved articles' bibliographies was undertaken and ten further articles of interest were identified. Twenty-two studies met the inclusion criteria (see Fig. 1 for flow chart of the search process).

The results of the search identified the literature relating to Pacific women, and the cervical screening pathway mainly relates to the cervical screening aspect. The synthesis of the information therefore mainly focuses on this aspect of the cervical screening pathway. Much of the literature comes from the United States and a small number of studies have been identified from New Zealand, Guam, American Samoa, and Fiji.

A critical appraisal was not undertaken of each article due to the variation of the articles and the inclusive character of a narrative review. The aim of this review was to synthesize the evidence in an attempt to understand the barriers and facilitators for Pacific women accessing the cervical screening pathway, which would enable the development of concepts and themes represented in the literature.

Data were extracted using a template adapted from a systematic review template.^14^ The following headings were used: study reference information, study design, participants and location, methods, intervention/outcomes, results summary, barriers, facilitators, notes, and comments. The first author carefully read the articles and made notes using the template designed to capture the relevant information.

Thematic analysis was utilized to determine the steps in the cervical screening pathway and the factors that worked to facilitate or hinder Pacific women's journey through the pathway. Each article was coded. From these, categories were developed. The first author then grouped relevant subthemes, ideas, and outcomes related to the barriers and facilitators for Pacific women. Even though data were extracted from a variety of sources using a narrative approach described by Pope and Mays,^16^ the data for analysis were compatible for integration into a “story telling approach” or single narrative synthesis.

Institutional review board approval was not required for this narrative review because it involved only a review of the literature.

## Results

Analysis showed the multifaceted and interrelated nature of the factors that impact on the cervical screening pathway. While the literature came from a variety health care environments, locations, and involved different groups of Pacific women or health care professionals, there were similar themes that came through in the thematic analysis.

### Cervical screening barriers

Barriers to cervical screening are multifaceted and the thematic analysis of the literature identified the following subthemes within the literature: cultural beliefs, fear, practical barriers, health care experience, knowledge, and education.

#### Cultural beliefs and attitudes

Cultural beliefs and attitudes have been identified as a barrier to cervical screening across the literature. The cultural belief that the lower genital tract is sacred and a part of the body to only be shared with husbands and no one else was prevalent in the literature.^17–25^ The topic of sex is taboo and not openly discussed by Pacific women with men or intergenerationally between younger and older women, making the discussion of cervical screening difficult for some Pacific women.^19,20,25,26^

Shame and stigma have been associated with cervical screening because undergoing a cervical smear is seen as an indication of a woman's inappropriate sexual behavior. This belief prevents some eligible women accessing cervical screening services.^17,19,22,23,26^ The cultural difficulty in discussing such issues and the associated stigma have been reported to affect discussions with health care providers because some Pacific women do not feel comfortable in raising their concerns or presenting for cervical screening.^17,23,26^

Preventative approaches to health screening are viewed differently to Western concepts of screening by Pacific women. Pacific women may not see the need for screening because they will often be asymptomatic and do not see the need to be tested or take time off work when they are not unwell.^19,20,23,25^ Cultural beliefs in regard to cervical cancer also influenced screening. Pacific women believe screening was no longer required once they had completed their childbearing as the use of the womb was no longer required.^20,23,27^

Competing priorities and responsibilities were identified throughout the literature. The collective nature of Pacific culture results in some Pacific women having many responsibilities to their family, both immediate and extended, community, and church. Such needs may take a higher priority to those of the woman's. Thus, it can make it difficult for some women to attend cervical screening and has been identified as a barrier.^18–20,24^

#### Fear

Fear of the unknown and the possibility of a cancer diagnosis or bad news have been identified as preventing women from undertaking cervical screening.^17,18,26,28^ Even when Pacific women identified as being knowledgeable about cervical screening, fear prevented them from being screened.^28^ The fear of pain or discomfort was also identified as a barrier.^28,29^

#### Practical barriers

Practical barriers are varied and include the cost of cervical screening both direct and indirect. The indirect costs such as time off work, transport, and childcare can prevent women from accessing cervical screening services.^19,20,28^ The literature from the United States identified that not having health insurance and the potential cost to family if cancer was detected were a barrier.^18,23,25^ In some cases, Pacific women's employment situation may hinder attendance due to having multiple part-time jobs. The socioeconomic status of Pacific women also impacts on their ability to access services as they often earn lower incomes, making it difficult to pay for care.^20,25^

#### Health care experience

Health care experience is an important aspect of engaging Pacific women and improving access to care and removing barriers. There were four main categories identified relating to health care experience, which included access to services, communication, health care provision, and confidentiality.

Hours of access and geographic locations were identified as barriers by both Pacific women and health care providers.^18,20,28,30^ Flexibility with service provision is an important aspect, given the competing priorities Pacific women manage. Newly immigrated women had difficulty accessing clinical services due to lack of knowledge regarding cervical screening and language.^25,28^ There is a lack of transparency regarding free cervical screening services, which could reduce the cost barrier for Pacific women.^20,28^ Conversely, a study within the Tongan community identified that participants did not utilize free services due to pride. In some cases, this meant they did not access any services.^25^

Staffs' poor communication, rudeness, inadequate information, and long waits in clinics have been identified as problematic and may negatively influence Pacific women's participation in screening.^23,28,31^ Pacific women have identified that health care professional's explanations are not always sufficient and some health care professionals do not listen to what they are saying.^20,28,31,32^ The importance of supporting Pacific women has been highlighted, that sometimes not pushing women into having a cervical smear test when they do not want to, instead building trust and rapport first may be the best way to engage Pacific women in the long term.^20^

Having a bad experience resulting in pain and discomfort sometimes resulted in some women not engaging in cervical screening.^17,20,24,28,29^ It was identified that there is a lack of Pacific health care providers and language-specific providers, which requires further development to improve access.^20,30^ However, it is recognized that Pacific women may prefer a non-Pacific health care provider due to concerns regarding confidentiality.^19,20,28^ Confidentiality of health care information is another key factor for Pacific women not accessing health care services and has been identified as a significant barrier to cervical screening if there is any concern regarding community gossip and the associated cultural stigma of cervical screening.^20,25,26,31^

Environment and facilities were identified as important when delivering cervical screening services and if insufficient were cited as a barrier. Ensuring privacy was a key element along with more practical aspects such as their bodies being covered, the use of plastic speculums rather than metal speculums, a comfortable examination bed, and a warm environment.^28,31^

#### Knowledge and education

Pacific women's knowledge of HPV and cervical cancer risk has been identified as limited. This lack of knowledge in turn influences their ability to access cervical screening services.^17,25,33–35^ DiStefano et al.^25^ reported that awareness in the Chamorro and Tongan communities about HPV was very limited. These studies highlight the need for further education and research into Pacific women's knowledge of HPV infection, particularly with the implementation of HPV vaccination programs and the proposed change from cervical cytology to primary HPV screening.

It has been identified that there is a lack of language-specific health information, which may be a barrier for some Pacific women. For newly immigrated Pacific women, there was a lack of resources available about cervical screening services.^25,28^

### Cervical screening facilitators

As with barriers to cervical screening, the facilitators are multifaceted and reflect what needs to change to remove the barriers Pacific women face. The thematic analysis identified a number of key subthemes that included the following.

#### Knowledge/education

Culturally tailored and language-specific programs are well received and improve cervical screening participation.^17–19,24,28,36,37^ The benefit of culturally tailored education sessions was that the information learnt was then disseminated into the community, to other family members, daughters, and nieces, particularly if the programs are supported by cultural and church leaders.^17,19,36,37^ Church settings have been identified as an opportune place to provide education to Pacific women. However, Mishra et al.^37^ reported that single Pacific women and those women who identified more closely with culture-specific beliefs had no increase in self-reported cervical screening following culturally tailored education. This raises the question whether church settings are the right place for single Pacific women due to the cultural taboos related to cervical screening.

Research has identified that health resources need to be language specific or in “plain” English and culturally appropriate to ensure information is accessible to Pacific women. Language-specific and culturally appropriate health resources can facilitate cervical screening through increasing knowledge.^18–20,27,29,38^ One study identified that native Hawaiian women who accessed health information via the Internet were more likely to participate in cervical screening, highlighting the various mediums for education.^35^

Two studies identified the importance of educating husbands in facilitating cervical screening.^17,39^ Providing a culturally appropriate approach to educating husbands is an important consideration in developing this strategy.^17^

Pacific women have identified that the use of Pacific radio and language-specific shows would be beneficial in improving cervical screening knowledge within their communities.^18,28^ It was suggested by Hawaiian women that advertising be extended to the mainstream media.^18^ A culturally appropriate advertising campaign in New Zealand targeting Pacific women saw a 12% increase in cervical screening coverage in Pacific women in the following 12 months, demonstrating the importance of tailoring media campaigns for Pacific women.^40^

#### Health care experience

Providing accessible health care services such as outreach, mobile clinics, and extended hours such as Saturdays and evenings facilitated screening.^18,19,28^ Transportation support also facilitated cervical screening for some women^19,28^ and free cervical screening services for Pacific women improved cervical screening.^28,36^ The availability of interpreters was identified as improving access for Pacific women.^28^

Pacific women were more likely to attend screening when there was a female provider.^17,19,28,36^ For young Pacific women in New Zealand, there was a strong preference to utilize family planning clinics. It is likely this provides a level of confidentiality for these young women; however, this has not been examined in the literature.^28^ This highlights the importance of having a number health care provider options available for Pacific women. Encouragement from health care providers, recalls, and reminders also assist in facilitating cervical screening.^27,28^ Pacific women are more likely to return for screening if they have had a positive experience.^20,28^

#### Culture

While cultural attitudes and beliefs may be a barrier, it is clear that it can also be a facilitator to screening. Pacific women have identified fear of cancer, peace of mind, and concern regarding protecting their family because there is a cultural responsibility that has facilitated their attendance for cervical screening.^28,29^ The support and encouragement of family were a predictor for cervical screening and may be reflective of culturally appropriate programs disseminating information and supporting other women.^18,28,41^

### Predictors for cervical screening

There were a number of studies that identified predictors for cervical screening and examined predictors against self-reported cervical screening (Table 2).

**Table 2.** Predictors for Cervical Screening in Pacific Women

Predictor for cervical screening	Source
Younger women	Sadler et al.,^27^ Balajadia et al.,^33^ Tran et al.,^35^ Tanjarsiri et al.,^41^ and O'Connor et al.^42^
Health insurance	Sadler et al.,^27^ Weiss et al.,^29^ Tanjarsiri et al.,^41^ Mishra et al.,^42^ and Mouttapa et al.^39^
Encouragement and advice from health care professionals	Tran et al.^35^ and Mishra et al.^42^
Recent clinical examination	Sadler et al.,^27^ Tran et al.,^35^ and Mishra et al.^42^
Support from family and friends	Tanjarsiri et al.^41^ and Mouttapa et al.^39^
Cervical cancer knowledge	Sadler et al.,^27^ Tanjarsiri et al.,^41^ Mouttapa et al.,^39^ and Tran et al.^35^
Formal education	Mishra et al.^42^ and Tran et al.^35^
U.S. born Pacific woman	Sadler et al.^27^

Studies in the United States identified health insurance as an important predictor for cervical screening. Two studies have reported that 81.7–86.7% of nonscreened women had health insurance, suggesting that insurance alone does not improve screening.^35,41^

### Colposcopy

There is a paucity of literature relating to Pacific women's experiences of colposcopy. One study by Adams and Ropiha^19^ undertook a qualitative evaluation of cervical screening education following the establishment of the NCSP in New Zealand. One component of this evaluation was an interview with a sole Pacific island community worker supporting Pacific women to colposcopy. There were a number of factors identified by the community worker preventing Pacific women attending their colposcopy appointments. They included the following: lack of education from health care professionals; fear and not understanding why they needed to attend; practical issues such as cost, transport, childcare, and having other priorities such as marriage difficulties. Supporting women to their appointments not only assisted with attendance but also provided cultural support. It was also highlighted that just sending women an appointment was not conducive for attendance. Contacting the women to arrange a suitable time for their appointment was a more preferable option.^19^ This provides some useful insight into the barriers Pacific women face accessing colposcopy services but does not examine Pacific women's experiences from their perspective. Further research is required in this area to understand the interface between primary and secondary care for Pacific women to reduce the inequity they face accessing colposcopy services.

## Discussion

This narrative review offers a unique perspective on the barriers and facilitators to cervical screening for Pacific women. These findings demonstrate that there are number of reasons Pacific women do not participate in cervical screening. Health care providers and policy makers need to take these into consideration when developing and providing cervical screening services to Pacific women. The facilitators for cervical screening provide guidance on how to improve cervical screening attendance for Pacific women.

Health promotion activities need to be culturally and linguistically appropriate for Pacific women and delivered in a number of ways, through either Church or community groups. This needs to be undertaken in collaboration with Pacific communities to ensure the success of such programs. However, there needs to be consideration for younger Pacific women and newly emigrated Pacific women as to how they access this information and what works for these groups; further research is required in this area. Given the stigma attached to cervical screening and lack of knowledge, further education of Pacific communities needs to be undertaken. However, further research is required in New Zealand to understand the extent of this issue.

Health care providers need to consider practical barriers such as cost, clinic environment and equipment, location of clinics, and hours of operation when providing services to Pacific women. They also need to support smear takers and consideration must be given as to how they enable staff to provide culturally competent care and service delivery, improving the experience for Pacific women. Health information needs to be linguistically appropriate and the use of interpreters must be available.

This review provides guidance for smear takers to minimize the barriers and facilitate cervical screening for Pacific women. For example, smear takers may be able to reduce the negative aspects of the cervical screening experience by providing culturally competent care, offering the option of a female smear taker, either Pacific or non-Pacific, ensuring confidentiality of health information, taking time with Pacific women to discuss what is involved, listening to their concerns and providing education, and ensuring that the clinical environment is conducive to a positive smear-taking experience. It is important for smear takers to discuss the options available to Pacific women such as family planning or other free smear-taking services when their own service may not meet the needs of Pacific women because this could reduce barriers.

While the literature explores the interplay between cultural beliefs and practices on Pacific women accessing cervical screening, there is no in-depth research in the field of colposcopy or from Pacific women's viewpoint. Research is required in this area given the significant delays Pacific women face accessing colposcopy services in New Zealand. While the barriers and facilitators to cervical screening may be similar, the experience of having an abnormality detected and colposcopy is different for women.^42,43^

There are limitations with this review, inherent with narrative reviews.^14,15^ One of the primary limitations was the inclusion of various study designs resulting in heterogeneity of the results. The thematic analysis while attempted in a nonbiased approach, there was the potential to lose richness of data in the process of synthesizing the data as some context and information may have been lost.^14,15^ However, while there are limitations, the narrative review was able to identify and summarize key barriers and facilitators specific to Pacific women that influence cervical screening attendance.

## Conclusion

Disparities accessing colposcopy clinics for Pacific women are significant in New Zealand and further research is required in this area. The cervical screening pathway extends from primary to secondary care and understanding the barriers and facilitators across the screening pathway is essential if we are to continue to reduce the disparities and cancer burden for Pacific women.

This review provides an important body of work with a more in-depth understanding of the cultural, practical, health care, and knowledge/education barriers and facilitators to cervical screening for Pacific women. Further research is required to understand Pacific women's knowledge of HPV and cervical screening, particularly with the move to primary HPV screening. Proactive strategies are required to tackle sociocultural attitudes, perceptions, and stigmatization toward cervical screening. Further understanding of how we educate our younger Pacific women also requires consideration.

## Abbreviations Used

HPV: human papilloma virus
NCSP: National Cervical Screening Programme
